# Repeat Closed-Head Injury in Male Rats Impairs Attention but Causes Heterogeneous Outcomes in Multiple Measures of Impulsivity and Glial Pathology

**DOI:** 10.3389/fnbeh.2022.809249

**Published:** 2022-03-11

**Authors:** Cole Vonder Haar, Sarah K. Wampler, Henna S. Bhatia, Jenny E. Ozga, Cory Toegel, Anastasios D. Lake, Christopher W. Iames, Caitlyn E. Cabral, Kris M. Martens

**Affiliations:** ^1^Injury and Recovery Laboratory, Department of Neuroscience, Ohio State University, Columbus, OH, United States; ^2^Injury and Recovery Laboratory, Department of Psychology, West Virginia University, Morgantown, WV, United States

**Keywords:** impulsivity, repeat brain injury, rat, concussion, CHIMERA

## Abstract

Repetitive mild traumatic brain injury, or concussion, can lead to the development of long-term psychiatric impairments. However, modeling these deficits is challenging in animal models and necessitates sophisticated behavioral approaches. The current set of studies were designed to evaluate whether a rubberized versus metal impact tip would cause functional deficits, the number of injuries required to generate such deficits, and whether different psychiatric domains would be affected. Across two studies, male rats were trained in either the 5-choice serial reaction time task (5CSRT; Experiment 1) to assess attention and motor impulsivity or concurrently on the 5CSRT and the delay discounting task (Experiment 2) to also assess choice impulsivity. After behavior was stable, brain injuries were delivered with the Closed-head Injury Model of Engineered Rotational Acceleration (CHIMERA) either once per week or twice per week (Experiment 1) or just once per week (Experiment 2). Astrocyte and microglia pathology was also assayed in relevant regions of interest. CHIMERA injury caused attentional deficits across both experiments, but only increased motor impulsivity in Experiment 1. Surprisingly, choice impulsivity was actually reduced on the Delay Discounting Task after repeat injuries. However, subsequent analyses suggested potential visual issues which could alter interpretation of these and attentional data. Subtle changes in glial pathology immediately after the injury (Experiment 1) were attenuated after 4 weeks recovery (Experiment 2). Given the heterogenous findings between experiments, additional research is needed to determine the root causes of psychiatric disturbances which may arise as a results of repeated brain injuries.

## Introduction

Traumatic brain injury affects over 2.8 million people in the United States annually (Center for Disease Control, 2019). This statistic merely reflects hospital visits and thus may not account for a significant additional group that does not seek treatment. Of the total injuries that happen every year, an estimated 70-90% fall into the mild (mTBI) category (Cassidy et al., 2004), with certain professions or activities such as sports at especially high risk (Rabadi and Jordan, 2001). Despite these often being considered “mild” at the time of injury, a significant subset of individuals goes on to experience enduring deficits. Notably, many of the associated behavioral impairments are within the social, emotional, and cognitive domains of function, commonly categorized as neuropsychiatric disturbances (Mahar et al., 2017).

In particular, repeat mTBI has come to be associated with chronic traumatic encephalopathy, a condition hallmarked by symptoms of behavioral disinhibition and increased emotional lability (Mahar et al., 2017). Some research has linked pathology such as tissue loss and tau deposition to these outcomes (McKee et al., 2016), yet these symptoms have been difficult to translate to animal models. Attempts have been made to model tauopathies that occur, but have had to rely largely on transgenic animals to achieve responses similar to those observed following human TBI (Yoshiyama et al., 2005; Ojo et al., 2016). Within functional studies of experimental TBI, one large focus, with some successes, has been on changes in the emotional response (Davies et al., 2016; Broussard et al., 2018), however, only one study has described changes in behavioral disinhibition following concussive injury (Mychasiuk et al., 2015).

A major problem that facing the experimental TBI field with regard to mTBI, is how to properly define “mild” in an animal model. There is debate over whether it should be limited by the total force applied, pathophysiological characteristics (e.g., little or no pathology), behavioral characteristics (e.g., little or no impairment), or some combination (e.g., no pathology, but functional impairment, or pathology but no functional impairment). In addition, there is discussion as to whether “milder” versions of more severe injury methods (e.g., controlled cortical impact, fluid percussion injury) are appropriate, or if mTBI should utilize only closed-skull models (Shultz et al., 2017). Recently, the Closed-Head Impact Model of Engineered Rotational Acceleration (CHIMERA) has been described in mice and rats. Injuries from the CHIMERA result in relatively subtle pathological (i.e., transient cytokine increase, increased glial reactivity, and minor white matter damage) and behavioral outcomes (i.e., transient sensorimotor and memory impairments), when repeat impacts are limited (Namjoshi et al., 2014, 2017). When additional injuries are given consecutively, stronger functional impairments such as working memory and choice impulsivity deficits emerge, but pathology is still limited (Nolan et al., 2018; Vonder Haar et al., 2019). However, when the injury severity is increased substantially, pathophysiological characteristics such as axonal injury, microglial activity, and cell death are amplified considerably (Sauerbeck et al., 2018; Bashir et al., 2020).

In human patients, neuropsychiatric complications are a major concern, yet the animal literature on mTBI is again limited in this regard. To model these types of behaviors in animals, sensitive functional assessments are necessary. Traditional cognitive assays such as the Morris water maze fail to capture deficits beyond basic learning and memory and are incapable of measuring psychiatric dysfunction. These issues are compounded by the fact that it is not clear whether identified deficits are due to generalized impairment in learning or more specific (e.g., memory) deficits. An alternate approach then would be to use stable assays of function, in which difficulty is sufficient to avoid floor or ceiling effects, or which represent a stable, trait-level preference of the animal. One behavior that is designed for difficulty and stable behavior is the five-choice serial reaction time (5CSRT) task. The 5CSRT task is capable of measuring attention and motor impulsivity within a single session, and can also collect some data on motivation, although that may be confounded with attentional deficits (Robbins, 2002). We have reported on this task in the context of focal TBI, and found that it was sensitive to even relatively mild injuries over an almost four-month testing period (Vonder Haar et al., 2016). The delay discounting task (DDT) represents another stable behavior in which choice impulsivity is assayed. This task assesses preference for a small reinforcer with a short (or no) delay versus a larger reinforcer with a much longer delay. We have previously shown this to be sensitive to repeat CHIMERA injuries (Vonder Haar et al., 2019).

The goals of the current report evolved across multiple studies. The first was to determine whether a rubberized tip would generate deficits on the 5CSRT after repeat injuries at two different frequencies (once/week or twice/week). When that revealed no deficits, a metal tip was used on those same rats with our previously published injury severity (Vonder Haar et al., 2019). Because it was not possible to dissociate the effects of the rubber from the metal tip, a second experiment was undertaken with just a metal tip. This second experiment sought to evaluate 5CSRT performance as before, but rats were also trained on the DDT task to obtain concurrent measurements of motor and choice impulsivity. Finally, histopathology of ventricle size, microglia activation and astrocyte activity were evaluated across both studies to determine whether functional impairments might be due to structural or pathological changes.

## Materials and Methods

### Overall Design

**FIGURE 1:**
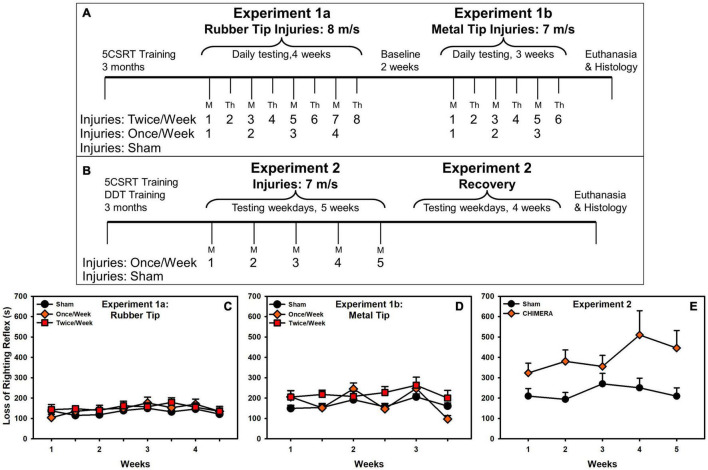
Experimental timeline and loss of righting reflex. **(A)** Experiment 1 timeline. **(B)** Experiment 2 timeline. **(C)** In Experiment 1a, there was no difference in LRR between groups. **(D)** In Experiment 1b, there was a significant interaction with Once/Week elevated on injuries (*p* = 0.044), and an overall increase for Twice/Week rats. **(E)** There was a significant LRR increase in CHIMERA-injured rats (*p* = 0.015).

Two experiments were undertaken. The first evaluated the effects of a rubberized injury tip, and when that failed to demonstrate any effects, a metal tip (Experiment 1a, 1b) was used. The primary behavioral outcome measures were motor impulsivity and attention. The second experiment used the metal tip and evaluated a new cohort of rats. The primary outcome measures were choice impulsivity, motor impulsivity, and attention. This experiment assessed a 4-week recovery period after injuries. Brains from both experiments were examined for glial pathology in response to the injury. Figures 1A,B presents the experimental timeline.

### Subjects

Male Long-Evans rats (*N* = 53) were restricted to 14 g chow daily with water available *ad libitum* (weights monitored weekly). Rats were pair-housed in standard rat cages (Experiment 1) or triple-housed in pentagonal OptiRat cages (Experiment 2; Animal Care Systems) on a reverse light cycle (12:12). Rats were approximately 2.5 months old when training began, and 5 (Experiment 1) or 6 (Experiment 2) months old at the first injury. Housing and procedures were approved by the West Virginia University Institutional Animal Care and Use Committee and performed in accordance with NIH’s Guide for the Care and Use of Laboratory Animals. Rats were divided across two experiments (Experiment 1, *n* = 30; Experiment 2, *n* = 23) to systematically evaluate the effects of tip type and impact velocity, injury frequency, and differing functional outcomes.

### Apparatus

Behavior took place in a bank of 16 operant chambers. The right wall of the chamber contained a pellet dispenser, two retractable levers with cue lights above them, and a house light at the top. The opposite wall consisted of 5 holes, all equipped with lights at the back that allowed for illumination and infrared beams for recording nose pokes to each. This study used the 5-hole array for the 5-choice serial reaction time task, and the levers for the delay discounting task. The pellet dispenser and house light were also used. Data were recorded using two PC computers running custom software programs written in MedPC-IV.

### Behavioral Training

In both experiments, rats underwent extensive operant behavioral training for a period of 3 – 4 months. Rats were matched for pre-injury performance and then randomly assigned to injury groups. Training and task details are provided below and within each experiment description. 5CSRT responses were recorded in nose poke holes and DDT responses were recorded on retractable levers.

#### Five-Choice Serial Reaction Time Task

Each trial began by a nose poke to the illuminated food hopper, which would initiate a 5 s intertrial interval (ITI), during which responses were required to be withheld. After the ITI, one of the five-choice holes, chosen at random, briefly illuminated for 0.5 s and rats had 5 s to make a response. Correct responses were reinforced with a single sucrose pellet. Incorrect (wrong hole), omitted (no response), or premature (response during ITI) responses were punished with a timeout and 5-s illumination of the house light. Full training parameters have been described elsewhere (Winstanley et al., 2010; Vonder Haar et al., 2016), but consisted of starting with a 30 s stimulus duration and gradually reducing the duration as rats achieve criterion responding, until they were able to complete the task at the final settings described above. Each session lasted for either 100 trials or 30 min.

#### Delay Discounting Task

An adjusting delay procedure was used as previously described (Evenden and Ryan, 1996; Vonder Haar et al., 2019) using retractable levers. Levers extended to start a trial and if no choice was made after 10 s, it was scored an omission. Choices on the “large” lever delivered 4 sucrose pellets after a delay, while choices on the “small” lever delivered 1 pellet immediately. Sessions consisted of 4 blocks of delays: 0, 10, 20, 40 s. Each block had 12 trials: 2 forced-choice on each lever (large/small), and 10 free choice. The intertrial interval was held constant at 48 s, minus any delay to reinforcement. Sessions lasted 48 trials and approximately 40 min.

### Closed-Head Impact Model of Engineered Rotational Acceleration (CHIMERA) Procedure

The CHIMERA device, which has been described in mice (Namjoshi et al., 2014, 2017) and rats (Vonder Haar et al., 2019) was used. CHIMERA delivers a focal impact to the closed skull with no incision, after which free head rotation is allowed, combining focal and acceleration forces. Anesthesia was induced with 5% isoflurane in a closed chamber (2 min), and the rat placed supine on the CHIMERA platform. A nose cone provided 2% isoflurane while two straps were adjusted at the rat’s midsection (20–30 s). The head was positioned such that the impact occurred approximately in front of bregma. In Experiment 1a, impact was delivered with a 100 g, 5 mm tip with a rubber cushion at a speed of 8 m/s (3.2 J). In subsequent experiments, impact was delivered with the same impactor, with the rubber tip removed, at a speed of 7 m/s (2.45 J, Experiments 1b, 2). The animal’s head freely rotated on impact.

Post injury, rats were injected subcutaneously with ketoprofen (5 mg/kg) and saline (5 mL), and placed in a heated recovery cage where loss of righting reflex (LRR) was recorded. The entire procedure lasted approximately three minutes. Shams received no impact, but were anesthetized and strapped into the CHIMERA device with anesthesia times yoked (matched) to injured rats. Anesthesia times were shorter in Experiment 1 (mean times across injuries 159 – 197 s) compared to Experiment 2 (mean times between 254 and 273 s), likely due to weight differences (Experiment 1: 400 g average; Experiment 2: 467 g average). This may have contributed to overall longer LRRs (Figures 1C-E).

### Histology

At the conclusion of behavior, rats were euthanized by transcardial perfusion with ice-cold phosphate-buffered saline, followed by 3.7% phosphate-buffered formaldehyde. Brains were then removed and placed in 3.7% phosphate-buffered formaldehyde for 24 h, then a 30% sucrose cryoprotectant solution. Brains were then embedded in a 15% gelatin matrix, and sliced, frozen, at 30 μm on a sliding microtome.

One series of sections was mounted to electrostatically-charged slides and stained with cresyl violet to inspect gross tissue loss. A series of three slices (−1.0, 0.0, 1.0 from bregma) were imaged on an Olympus microscope (BX-43) and camera (DP-80, 12.5 megapixel) at 1.25X magnification and the area of the ventricles measured using ImageJ (NIH, Bethesda, MD, United States). These areas were used to estimate ventricular volume by multiplying the average area by the distance.

A second series of slices was stained with IBA1 to identify microglia. Slices were blocked in normal goat serum for 8 h, then incubated with rabbit anti-IBA1 primary antibody (Wako-Chem 019-19741; 1:2000) for 24 h, rinsed, and then incubated in biotinylated goat anti-rabbit IgG secondary antibody (Vector, BA-1000; 1:2000) for 2 h, rinsed, and then reacted with an avidin-biotin complex kit (Vectastain PK-6100) and catalyzed with 0.05% diaminobenzidine and 0.15% hydrogen peroxide.

A third series of slices was stained with GFAP to identify astrocytes. Slices were blocked in normal goat serum for 8 h, then incubated with anti-GFAP primary antibody (Abcam AB7260; 1:3000) for 48 h, rinsed, and then incubated in biotinylated goat anti-rabbit IgG secondary antibody (Vector, BA-1000; 1:2000) for 2 h, rinsed, and then reacted with an avidin-biotin complex kit (Vectastain PK-6100) and catalyzed with 0.05% diaminobenzidine and 0.15% hydrogen peroxide.

For IBA1 and GFAP stains, five regions of interest (ROIs) were selected: medial prefrontal cortex (mPFC), orbitofrontal cortex (OFC), dorsal striatum (dSRT), nucleus accumbens core (NAc-Core), nucleus accumbens shell (NAc-Shell; Experiment 1), and hippocampus (CA3; Experiment 2). Hippocampal slices were not taken for Experiment 1. Three images from each ROI were taken with a 40x magnification objective. GFAP cell counts were performed manually, blinded to group condition, by a primary rater and verified by a second rater using ImageJ (NIH, Bethesda, MD). To assess total astrogliosis, ImageJ was used to calculate the percent area occupied by GFAP^+^ staining. Microglia cell counts were performed automatically using ImageJ’s “count particles” feature, with minimal size definitions. To assess microglia phenotype, automated counts were also performed, but ImageJ’s “circularity” parameter was systematically increased (0, 0.10, 0.15, 0.20, 0.30) to limit counts to more activated, circular, amoeboid-like, microglia.

### Experiment 1

Rats were trained on the 5CSRT task until they reached a stable baseline (56 sessions). Rats were tested during the weekdays in training, and seven days a week during testing. Upon reaching baseline, rats were injured using CHIMERA and re-assessed as described below. Rats were assigned to one of three groups: sham (*n* = 10), one injury per week (Once/Week, *n* = 10), and two injuries per week (Twice/Week, *n* = 10). Groups were kept the same through Experiment 1a and Experiment 1b. The Once/Week group received injuries Mondays and sham procedures Thursdays, while the Twice/Week group received injuries both days.

#### Experiment 1a

The first experiment evaluated the use of a rubberized tip because recent studies report utility for modeling concussion with this type of impact (Shitaka et al., 2011; Broussard et al., 2018). Rats were 5 months old at first injury and underwent injuries for 4 weeks. Between injuries, behavior was assessed daily. At the conclusion of the four weeks, behavioral challenges were administered to determine whether increasing task difficulty could elicit injury-induced deficits (see Supplement for methods and results). There was no difference between the groups, thus the same rats were used for Experiment 1b.

#### Experiment 1b

The second experiment evaluated injuries delivered with just a metal tip, as described in prior CHIMERA studies (Namjoshi et al., 2014, 2017; Vonder Haar et al., 2019). Groups were the same as Experiment 1. Baselines were verified as stable with two weeks of behavior. Impact velocity was reduced to 7 m/s out of concerns over skull fracture (based upon cadaver pilots). Rats were 6.25 months of age at the start of the three weeks of injuries and behavior was conducted as described above. At the conclusion, rats were given four sessions of progressively easier stimuli durations to test general visual function (see Supplement for methods and results).

### Experiment 2

Rats were concurrently trained on the 5CSRT and the Delay Discounting Task (DDT) until they reached a stable baseline (77 sessions 5CSRT, 57 sessions DDT). 5CSRT was conducted in the morning and DDT in the afternoon. Rats received half measures of food after each task. To our knowledge, this is the first report of such concurrent training in the same cohort of animals. Rats were tested during the weekdays. Upon reaching baseline, rats were assigned to CHIMERA (7 m/s; *n* = 13) or Sham (*n* = 9) groups. Rats were injured weekly for 5 weeks and then assessed for four weeks of post-injury recovery. Injuries occurred on Mondays. Part way through the experiment, a user error occurred with the DDT parameters, resulting in 7 rats receiving a different set of delays (0, 5, 10, 20 s) after injuries 3-5. To address this, the k-value, which is stable across delays, was used in calculating choice impulsivity.

### Data Analysis

The primary 5CSRT variables of interest were Attention (percent correct), Motor Impulsivity (percent pre-matures), Motivation (percent omissions), and the task efficacy index (Correct/[Incorrect + Pre-matures + Omissions]). The efficacy index is used to give a global view of deficits on this task (Vonder Haar et al., 2016). The number of trials, latency to respond, and latency to collect the reinforcer were also collected (see Supplementary Material). The primary DDT variables of interest were Motivation (percent omissions) and Choice Impulsivity which was calculated by recording the percent large choice across the set of delays and fit to a hyperbolic equation: 1/(1 + k*Delay). The decay rate of that curve, *k*, was then analyzed. Latencies to respond were also collected.

Statistical tests were conducted using R statistical software^1^, using the *stats*, *lme4*, and *lmerTest* libraries. Repeated measures data (behavioral variables, loss of righting, histology) were analyzed using mixed effects regression, with rat intercepts as the random effect, and baseline performance as a covariate. Behavior sessions were nested within bins of 3- or 4-days post-injury for Experiment 1, and week for Experiment 2 (see figures). Ventricle volumes were compared in a one-way ANOVA or *t*-test. Transformations were applied to data to normalize distributions: log transformation for data bounded on the lower spectrum and ratio data (collection and choice latencies, task efficacy index, omissions, pre-matures, *k* values) and the arcsine-square root transformation was used for percentage variables bounded on the upper end (accuracy). A *p*-value of less than 0.05 was considered significant. Principal components analysis was used to identify common variance in behavioral tasks for Experiment 2.

## Results

Additional data, including tables of statistical results are available in the supplement.

### Mortality (All Experiments)

Across the studies, mortality was moderate. In Experiment 1a, no deaths occurred with the rubber tip. In Experiment 1b, only one death occurred after five injuries with the metal tip (Twice/Week group). In Experiment 2, three deaths occurred: one immediately after 3 injuries, one found dead next day after 3 injuries, one euthanized due to poor recovery after 4 injuries. Total death rate was 12% in CHIMERA groups across the two studies, or 3% per metal tip injury.

### LRR (All Experiments)

Results are presented in Figures 1C–E. There were no effects in Experiment 1a, but Experiment 1b revealed an interaction (*F*(2, 146.31) = 3.20, *p* = 0.044) such that, relative to sham, Once/Week rats had elevated LRRs on injury days and Twice/Week had overall elevated LRRs. In Experiment 2, there was an overall increase in LRR due to injury (β = 0.89, *t* = 2.48, *p* = 0.015).

### Experiment 1a: Weekly or Biweekly 8 m/s CHIMERA (Rubberized Tip) on 5CSRT

**FIGURE 2:**
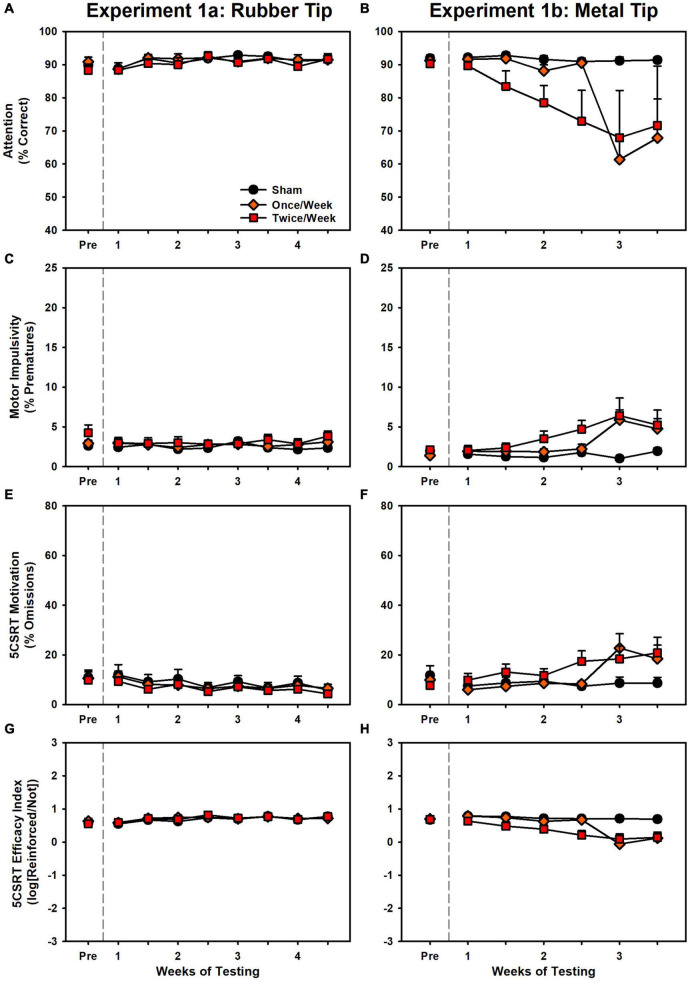
Performance on the 5CSRT during Experiment 1a (left panels) and Experiment 1b (right panels). **(A)** There were no group differences in accuracy. **(B)** There was a significant decrease in accuracy in Once/Week and Twice/Week groups relative to sham (*p* < 0.001, *p* < 0.001). **(C)** There were no group differences in pre-matures. **(D)** There was a significant increase in pre-matures in Once/Week and Twice/Week groups relative to sham (*p* < 0.001, *p* < 0.001). **(E)** There was a small but significant decrease over time in the Twice/Week group relative to sham (*p* = 0.003). **(F)** There was a significant increase in omissions in Once/Week and Twice/Week groups relative to sham (*p* < 0.001, *p* < 0.001). **(G)** There were no group differences in the task index. **(H)** There was a significant decrease in the task index in Once/Week and Twice/Week groups relative to sham (*p* < 0.001, *p* < 0.001).

Results are presented in Figure 2 and Supplementary Figure 1. There were no significant differences in accuracy, pre-matures, total trials, or latency to collect the reinforcer (*p*’s > 0.094, Supplementary Table 1). However, small (β’s < 0.11), but significant differences (*p*’s < 0.023, Supplementary Table 1) were detected in omissions, efficacy index and response latency in the direction of *improvement* for the Twice/Week group. The lack of impairment is starkly contrasted with the detrimental effects of successive injuries observed in Experiment 1b. A set of behavioral challenges were conducted to determine if any subtle effects occurred due to the injuries (see Supplement). There were no effects for these behavioral challenges (Supplementary Figure 2 and Supplementary Table 3).

### Experiment 1b: Weekly or Biweekly 7 m/s CHIMERA (Metal Tip) on 5CSRT

Results are presented in Figure 2 and Supplementary Figure 1. There were significant effects of injury for every variable: accuracy, pre-matures, omissions, efficacy index, total trials, response latency, and reinforcer collection latency (*p*’s < 0.025, Supplementary Table 2). Once/Week and Twice/Week animals were significantly impaired relative to Sham on every variable (*p*’s < 0.002), but there were no differences between the two injury groups (*p*’s > 0.318). Rats that performed poorly at the conclusion of the study were evaluated on progressively easier versions of the 5CSRT as a proxy to determine visual function (see Supplement). There were impairments even with long stimulus durations (Supplementary Figure 3 and Supplementary Table 4).

### Experiment 2: Weekly 7 m/s CHIMERA (Metal Tip) on 5CSRT + DDT

**FIGURE 3:**
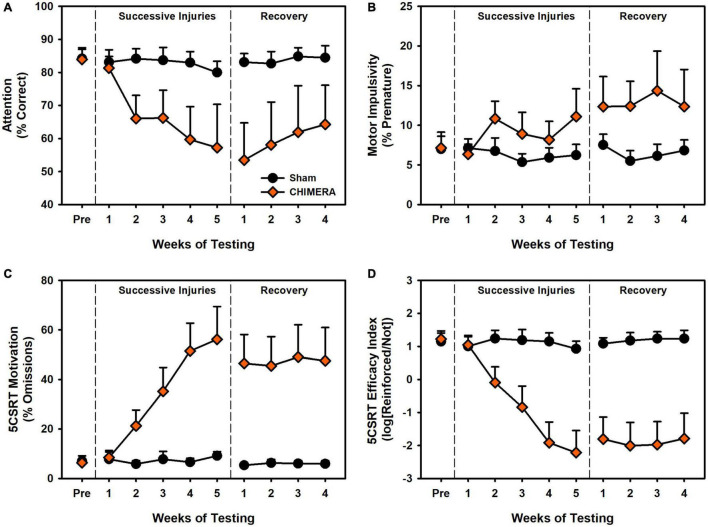
Performance on the 5CSRT during Experiment 2. **(A)** There was a significant decrease in accuracy over repeated injuries (*p* < 0.001) which persisted into the recovery period (*p* = 0.004). **(B)** There was a significant increase in pre-matures over repeated injuries (*p* < 0.001) however this was no longer significant in the recovery period (*p* = 0.955). **(C)** There was a significant increase in omissions over repeated injuries (*p* < 0.001) which persisted into the recovery period (*p* = 0.006). **(D)** There was a significant decrease in the task index over repeated injuries (*p* < 0.001) which persisted into the recovery period (*p* < 0.001).

**FIGURE 4:**
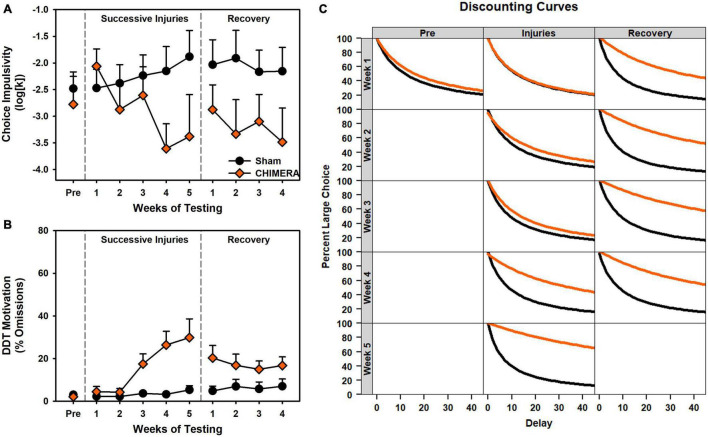
Performance on the DDT during Experiment 2. **(A)** There was a significant decrease in *k* values over repeated injuries (*p* < 0.001) which persisted into the recovery period (*p* = 0.045). **(B)** There was a significant increase in omissions over repeated injuries (*p* < 0.001) which persisted into the recovery period (*p* = 0.027). **(C)** Curves of the fitted discounting data from the *k* value.

Results are presented in Figures 3, 4. During the injury period, there was significant decrease in 5CSRT performance for CHIMERA animals in accuracy, omissions, efficacy index, total trials, and response latency (*p*’s < 0.001; Supplementary Table 5). However, there was no impairment in pre-matures or reinforcer collection latency (*p*’s > 0.466; Supplementary Table 5). During the recovery period, these CHIMERA-induced impairments persisted across the same measures (*p*’s < 0.006; Supplementary Table 5).

For the DDT, there was a significant decrease in *k* value over time (i.e., reduced impulsivity) for the CHIMERA rats (*p* < 0.001; Supplementary Table 6). For omissions and choice latency during the injury period, there was an Injury x Week x Delay interaction (*p*’s < 0.006; Supplementary Table 6) such that CHIMERA rats increased over time and delay relative to Sham (β’s > 0.06; Supplementary Table 6). All of these deficits continued into the recovery period (*p*’s < 0.045; Supplementary Table 6).

### Histopathology (All Experiments)

#### Microglia Activity

**FIGURE 5:**
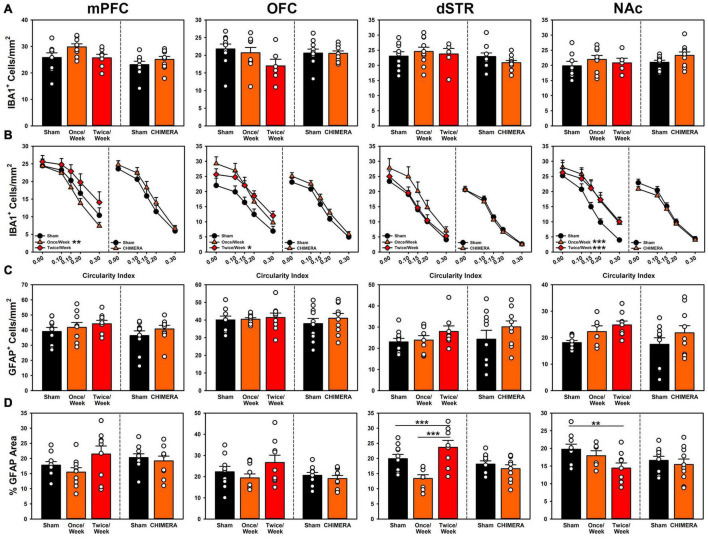
Histopathology results from both experiments across (left to right) medial prefrontal cortex, orbitofrontal cortex, dorsal striatum and nucleus accumbens. In each panel, the left side of the dividing line is Experiment 1, and the right side Experiment 2. **(A)** Total microglia counts were not significantly different for any region for either experiment. **(B)** Microglia phenotypes for Experiment 1 were significantly more reactive for the Once/Week in PFC and NAc (*p* = 0.006, *p* < 0.001) and for the Twice/Week in OFC and NAc (*p* = 0.023, *p* < 0.001). Phenotypes remained unchanged for injuries in Experiment 2. **(C)** Total astrocyte counts for Experiment 1 were significantly elevated for the Twice/Week group relative to sham (*p* = 0.039), but not differential by ROI. There were no changes in Experiment 2. **(D)** Gliosis in Experiment 1 was significantly decreased for Once/Week rats in the dSTR (*p* < 0.001) and for Twice/Week rats in the NAc (*p* = 0.010). There were no changes in Experiment 2.

Results are presented in Figures 5A,B. For Experiment 1, There was an overall Group*ROI effect (*p* < 0.001). However, when individual regions were compared, there were no significant group differences in IBA1^+^ cell counts at any ROI (Supplementary Table 7), although the Once/Week group approached significance in the OFC (*p* = 0.053), and the Twice/Week group approached significance in the dSTR (*p* = 0.053). For microglia phenotype, the Once/Week group had fewer reactive microglia in the PFC (*p* = 0.006), but more in the OFC and NAc (*p’s* < 0.025) and the Twice/Week group had increased reactive microglia in the OFC and NAc (*p*’s < 0.023). For Experiment 2, there were no injury-related changes in microglia count across the ROIs (*p*’s > 0.053). There were significant Group*ROI differences when examining microglia phenotype (*p* = 0.001), however since there were no interactions with phenotype (Supplementary Table 8), this was merely due to the increased power when quantifying at multiple levels of cell circularity. See supplement for all statistical comparisons.

#### Astrocyte Activity

Results are presented in Figures 5C,D. For Experiment 1, there was a global increase in GFAP^+^ cell counts across all areas for the Twice/Week group compared to sham (*p* = 0.039), but no other effects (Supplementary Table 7). Overall astrogliosis as percent GFAP^+^ area was reduced in Once/Week animals in the dSTR (*p* < 0.001) and reduced in the Twice/Week animals in the NAc (*p* = 0.010) compared to Sham. For Experiment 2, there were no injury-related changes in astrocyte cell count or total gliosis (*p*’s > 0.801; Supplementary Table 8). See supplement for all statistical comparisons.

#### Tissue Loss

**FIGURE 6:**
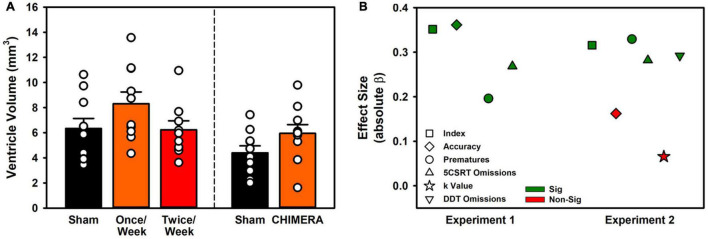
Ventricle volume and relation between LRR and behavioral variables. **(A)** Ventricle volumes were unchanged in Experiment 1, but repeat CHIMERA significantly enlarged ventricles in Experiment 2 (*p* < 0.001). **(B)** Loss of righting significantly predicted impairment for 8/10 measured variables across the two experiments.

For Experiment 1, The ventricle volume was compared in a one-way ANOVA (Volume ∼ Group). There was no significant difference between the groups (*F*_(2,28)_ = 1.13, *p* = 0.340; Figure 6A). For Experiment 2, a t-test was performed. Rats in the CHIMERA group had significantly higher ventricular volumes relative to sham (*t*_(18)_ = 4.82, *p* < 0.001; Figure 6A).

### Loss of Righting Reflex as Predictor of Impairment (All Experiments)

To determine whether the LRR could be used to predict behavioral impairment, a series of regressions were performed. Data were isolated for injury events and the resulting week or half-week of behavioral testing (i.e., no sham rats, and only injury time points from Experiment 1 Once/Week rats). For Experiment 1, LRR was predictive of performance on index, accuracy, omission, and premature variables (*p*’s < 0.001). For Experiment 2, only accuracy and the choice impulsivity *k* value were not predicted by LRR (*p*’s < 0.005; Figure 6B and Supplementary Table 9).

### Relation Between Choice and Motor Impulsivity (Experiment 2)

**FIGURE 7:**
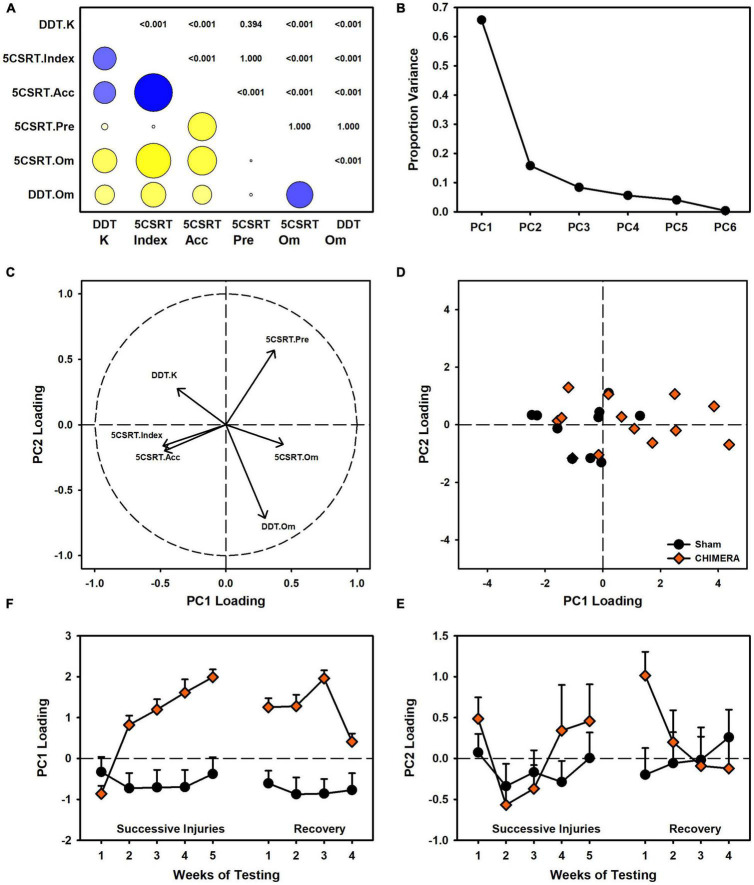
Relation between 5CSRT and DDT performance in Experiment 2. **(A)** Other than premature responses, many behavioral variables were significantly correlated (blue = positive, yellow = negative, circle size indicates strength, digits indicate adjusted *p* values). **(B)** A principal components analysis identified two major components accounting for 81.5% of variance in behavior. **(C)** Component loadings indicated similarities between index and accuracy, as well as both omission measures. *K* values were orthogonal to other measures. **(D)** CHIMERA animals were right-shifted on PC1 but had similar spread to shams on PC2. **(E)** Plotting of PC1 loading over time revealed strong shifts due to the injuries. **(F)** PC2 showed no group-related changes over time.

The data from experiment 2 were examined to better understand the discrepant relationship between choice and motor impulsivity outcomes. A set of correlations (with Bonferroni correction) was performed between all major behavioral measures: 5CSRT index, accuracy, pre-matures, and omissions and DDT *k* value and omissions. There were strong correlations between most measures, with the exception of pre-matures vs. omissions, index, and k (Figure 7A). To further understand these relations, a principal components analysis was performed, revealing two major components accounting for 65.7% and 15.8% of the variation in behavioral data (Figure 7B). Component loadings revealed similar strong loadings for accuracy and index, and similar directional loading for both omission measures. Interestingly, *k* values and pre-matures were nearly orthogonal across the two components (Figure 7C). Injury-related changes in loading strength on these components were primarily distributed across PC1 (Figures 7D,E).

## Discussion

The study of mTBI is one of the most difficult topics in the field of neurotrauma. By definition, mTBI results in relatively minor functional deficits, often only enduring in a select subset of individuals (Petraglia et al., 2014a; Shultz et al., 2017). Thus, to investigate these changes, new approaches in methodology are required. In the current study, we combined a model of repeat head injury, the CHIMERA, with sensitive behavioral tests, and identified heterogenous effects on attention and impulsivity after repeat mTBI. Specifically, both attention and motor impulsivity were impaired after repeat injury with a metal tip (Experiment 1b), but a replication of these effects was not precise (Experiment 2) and revealed a large deficit in attention which was persistent, but no impairment in motor or choice impulsivity. Further complicating the interpretation of these mTBI data was the pathophysiology in the current study. These data revealed subtle changes in glial phenotypes after injury, but only in rats from Experiment 1 which had the additional rubber tip injuries and were euthanized shortly after their final injury. More surprising was the small, but significant increase in ventricle size from rats in Experiment 2 which were not euthanized until four weeks after the final injury. Together, these data highlight the difficulty of establishing consistent functional deficits and directly linking them with pathological outcomes.

Despite the growing body of work on experimental concussion, there is considerable heterogeneity in the literature. For instance, a number of studies have observed only transient deficits after mTBI (Rostami et al., 2012; Marschner et al., 2018), or have required a large number of injuries (Petraglia et al., 2014b; Nolan et al., 2018). In addition, there is considerable variation even within similar models. Prior studies have reported on impairments after the use of a rubberized impact tip (Shitaka et al., 2011; Broussard et al., 2018), but we observed no deficits even after 8 rubber tip injuries (Experiment 1 and Figure 2). Moreover, although we have previously established operant behavioral methods as sensitive and reliable for detecting injury-induced deficits (Vonder Haar et al., 2016; Scott and Vonder Haar, 2019; Shaver et al., 2019), even with the CHIMERA model (Vonder Haar et al., 2019), the DDT results from Experiment 2 (Figure 4) were in the opposite direction of our previous report. Even within the current set of experiments, conducted with the same laboratory personnel and equipment, there were differences in 5CSRT performance, particularly in omissions and the overall efficacy index variables (Figures 2, 3; scaled identically for ease of comparison).

Pathological data offer similar concerns over heterogeneity and identification of relevant pathophysiological characteristics. Studies of chronic outcomes in repeat mTBI patients have revealed ongoing psychiatric dysfunction, and some researchers have linked these behavioral outcomes to pathological features such as tau accumulation (McKee et al., 2016; Mahar et al., 2017). However, these pathologies have been difficult to replicate in non-transgenic animal models. Instead, much of the focus has been placed on markers of inflammation and glial malfunction. Prior studies with the CHIMERA platform have identified subtle effects, including transient increases in cytokines and microglia activity (Namjoshi et al., 2014, 2017), and dopaminergic dysfunction (Vonder Haar et al., 2019). In the current study we similarly observed relatively minor changes in glial reactivity. In Experiment 1, absolute microglia numbers were not changed, but a potentially more proinflammatory phenotype of amoeboid microglia were identified across multiple regions. These did not persist to the 4-week post-injury time point measured in Experiment 2 (Figure 5B). However, ventricle volumes were increased (Figure 6A), suggesting that although microglia had normalized by then, there may be ongoing neurodegeneration. Other data identify degeneration more than one month post-CHIMERA along the optic tracts, an area that likely experiences maximal rotational forces (Tucker et al., 2021).

Notably, these minor differences in pathological data between the two experiments may also contribute to the observed functional differences. For instance, in Experiment 1, LRR deficits were smaller in magnitude (Figure 1; although likely due to anesthesia times) and omissions were substantially smaller for Experiment 1. While these may merely reflect sampling error due to variance between cohorts, it is possible that there may have been some level of preconditioning due to the rubberized impacts experienced by the rats in Experiment 1. Other researchers have noted that low-dose lipopolysaccharide may result in neuro- and functionally-protective effects (Longhi et al., 2011; Turner et al., 2017; Eslami et al., 2020). Given the slightly changed microglial phenotype, there may have been some protective response. This may be further supported by the sudden drop in Once/Week rats after the third injury as opposed to the more progressive pattern observed in the Twice/Week group (Figure 2). Despite this, caution should be used in interpreting such data as other researchers have observed exacerbated responses (i.e., priming) to inflammatory challenge after a brain injury (Fenn et al., 2014; Kaukas et al., 2021). However, it should be noted that for the lone study which used an easier variant of the 5CSRT task, this was limited to omissions (Kaukas et al., 2021). Additional studies have indicated that “subconcussive” impacts may also generate pathology and ultimately functional impairment if repeated frequently enough (Lavender et al., 2020; Hiles-Murison et al., 2021).

Given the heterogeneity in functional and pathological outcomes for experimental concussion described above, and to improve injury modeling, a particular effort should be made to identify replicable outcomes. Several features are consistent across the literature and may inform the current findings. While the histological findings from the current study were relatively minor and limited to the acute time points, they are not entirely surprising, given that prior studies with the CHIMERA platform have identified relatively subtle markers (Namjoshi et al., 2014, 2017; Vonder Haar et al., 2019; Tucker et al., 2021). One potential explanation linking these relatively minor pathological features and substantial dysfunction lies at the level of cortical circuits. Complex behaviors such as the 5CSRT task and DDT integrate large forebrain circuits (Robbins, 2002), and it would not be particularly surprising if impaired communication between these regions was the ultimate cause of behavioral symptoms after repeat concussions. Glial pathology in these regions may contribute to the evolution of dopaminergic deficits which have previously been identified as likely contributors to dysfunction (Vonder Haar et al., 2019). Medial prefrontal circuits may be particularly relevant in the case of the small-magnitude increases in motor impulsivity which were significant in Experiment 1 due to very tight variability, but insignificant in Experiment 2. Few other studies have used operant behaviors to assess experimental concussion. However, the existing studies also evaluate frontal-dependent measures. A study by Mychasiuk et al. (2015) revealed inhibitory deficits after a single juvenile concussion using a go/no-go task. Nolan and colleagues identified deficits in working memory after repeat CHIMERA injury linked to changes in neuronal excitability in the prefrontal cortex (Nolan et al., 2018).

The current study and existing literature provide a strong argument for sophisticated functional assessments to better understand repetitive brain injury. Behavioral tasks and batteries designed to pull apart unique contributions of various circuits combined with targeted pathology may provide the best avenue for establishing causes and treatments for functional deficits associated with repeat injury. In the case of rotational injuries in the rodent such as the CHIMERA, maximal rotational forces will be concentrated across the frontal region as that is furthest from the axis of rotation. Indeed, we see deficits that, at least partially, align with that. However, in the current data, choice impulsivity data changed in a direction opposite of motor impulsivity, and an analysis of these data together suggested the two measures were very separate (Figure 7). Importantly, these data were influenced by the injury itself (Figures 7D–F) and so may not be reliable when considering impulsivity in intact animals. Of concern for the CHIMERA model is the potential for visual deficits after injury. Tucker and colleagues reported visual trial deficits in the water maze concurrent with optic tract damage (Tucker et al., 2021), which could account for the attentional and even motivational deficits observed in the current study and potentially even the unexpected changes in choice impulsivity given the high omissions in that task (Figure 4). A highly-variable response to optic damage could also explain discrepancies between the experiments in 5CSRT task omissions (Figures 2F vs. 3C). Moreover, this may also explain why CHIMERA rats in Experiment 2 omitted at higher rates on 5CSRT (peak 56.2%) versus DDT (peak 29.8%) behaviors, since the DDT presents levers which audibly extend to a fixed location, making DDT less visually-dependent (Figures 3C vs. 4B). Given the numerous tasks which rely on vision in the rodent, this should be strongly considered in selection and design of assessment measures and specific acuity evaluations used to rule out visual impairment.

In sum, although deficits from the current study were heterogenous, this may be explained by some combination of impairments in vision and alterations to frontal circuits. Further, glial pathology was only observed acutely, but at one month post-injuries transitioned to minor levels of general atrophy as evidenced by increased ventricles. The use of operant behaviors such as the ones described here enable researchers to establish strong baselines and adjust the number of injuries based on the magnitude of deficits. Experiment 1 provides an example of this – when the rubber tip did not cause deficits, we were able to adapt to a metal tip and reevaluate a second course of injuries. However, caution should be taken on tasks which require visual integrity given the current findings and reports from recent labs over visual impairment. Ultimately, improving behavioral assessments for repeat TBI may allow us to better understand the underlying pathophysiology and lead to identification of treatments.

## Data Availability Statement

The raw data supporting the conclusions of this article will be made available by the authors, without undue reservation.

## Ethics Statement

The animal study was reviewed and approved by West Virginia University IACUC.

## Author Contributions

CV and KM contributed to the concept and design of the experiments. SW, HB, JO, CT, AL, CI, and CC contributed to data collection. CV, KM, SW, and JO contributed to data analysis. CV, KM, SW, HB, and JO contributed to writing. All authors contributed to the article and approved the submitted version.

## Conflict of Interest

The authors declare that the research was conducted in the absence of any commercial or financial relationships that could be construed as a potential conflict of interest.

## Publisher’s Note

All claims expressed in this article are solely those of the authors and do not necessarily represent those of their affiliated organizations, or those of the publisher, the editors and the reviewers. Any product that may be evaluated in this article, or claim that may be made by its manufacturer, is not guaranteed or endorsed by the publisher.

## Footnotes

1
http://www.r-project.org/
