# Supplementary material for: Repeat Closed-Head Injury in Male Rats Impairs Attention but Causes Heterogeneous Outcomes in Multiple Measures of Impulsivity and Glial Pathology
Source: Front Behav Neurosci. 2022 Mar 11;16:809249. doi: 10.3389/fnbeh.2022.809249 (PMC8963781; doi:10.3389/fnbeh.2022.809249)
Supplement: Supplementary file 1 [file Data_Sheet_1.docx]

**SUPPLEMENTAL MATERIAL**

**Repeat Closed-Head Injury in Male Rats Impairs Attention but Causes Heterogeneous Outcomes in Multiple Measures of Impulsivity and Glial Pathology**

**Methods**

**Experiment 1a: Impulsivity and Attention Challenges**

At the conclusion of the four weeks, behavioral challenges were administered to determine whether increasing task difficulty could elicit injury-induced deficits. Accuracy and premature challenges were conducted during two separate sessions. During the accuracy challenge, the stimulus time (hole illumination) was decreased from 0.5 s to 0.25 s. For the premature challenge, the ITI, where responses must be withheld, was increased from 5 to 7.5 s.

**Experiment 1b: Stimulus Duration Manipulation**

Because severe deficits emerged, at the conclusion, rats were assessed on a progressively easier version of the task (stimulus durations: 1, 2.5, 5, 10 s) across sessions. This determined whether we were selective cognitive dysfunction, or if symptoms had emerged due to other problems such as visual impairment.

**Results**

**Experiment 1A: 5CSRT Impulsivity and Attention Challenges**

Data from the two challenges were analyzed in a one-way ANOVA (*Outcome* *Variable* ~ Group; Figure S2). For the accuracy challenge, the target stimulus was decreased from 0.5 to 0.25 seconds. While this decreased performance overall, there was no difference between the three groups on the primary variables (Accuracy: *F*(2, 26) = 0.60, *p* = 0.554; Prematures: *F*(2, 26) = 0.26, *p* = 0.775; Omissions: *F*(2, 26) = 0.34, *p* = 0.715). For the premature challenge, the intertrial interval was increased from 5 to 7.5s and once again performance was impaired, but in a uniform manner across groups (Prematures: *F(*2, 25) = 0.38, *p* = 0.687; Accuracy: *F*(2, 25) = 0.80*, p* = 0.461; Omissions: *F*(2, 25) = 0.75, *p =* 0.484). These results confirmed that even when challenged, repeat TBI with the rubber tip caused no deficits in attention and impulsivity.

**Experiment 1B: Stimulus Duration Manipulation**

Data were analyzed in a linear mixed effects regression (*Outcome Variable* ~ Group*Stimulus Duration; Figure S3). Any rats that could not perform the task at easier settings (suggesting more global deficits rather than selective cognitive dysfunction) had their data re-examined and time points where overall performance dropped to chance levels (once/week = 2, twice/week = 4) were removed from all described analyses in the main document. One advantage of mixed effects models is that listwise deletion is not needed for these cases. Had they remained in the analysis, it would not be known if deficits were selective to impulsivity or attention, or if they were more global in nature (e.g., visual or motivational impairment). There was a significant main effect of group on accuracy (*F*(2, 20.98) = 7.07, *p =* 0.005), such that both the Once/Week and Twice/Week groups were significantly more impaired than the Sham group across all stimulus levels (Once/Week: β = -1.45, *t =* 3.61, *p =* 0.002; Twice/Week: β = -1.15, *t =* 1.18, *p =* 0.026). There was also a significant main effect of increasing the stimulus duration in that it increased accuracy, but there was no interaction between group and stimulus duration (see Table S4 for a full summary). However, overall group differences disappeared for omitted responses and only approached significance for prematures (*p* = 0.075) under the stimulus duration manipulation (see Table S4).


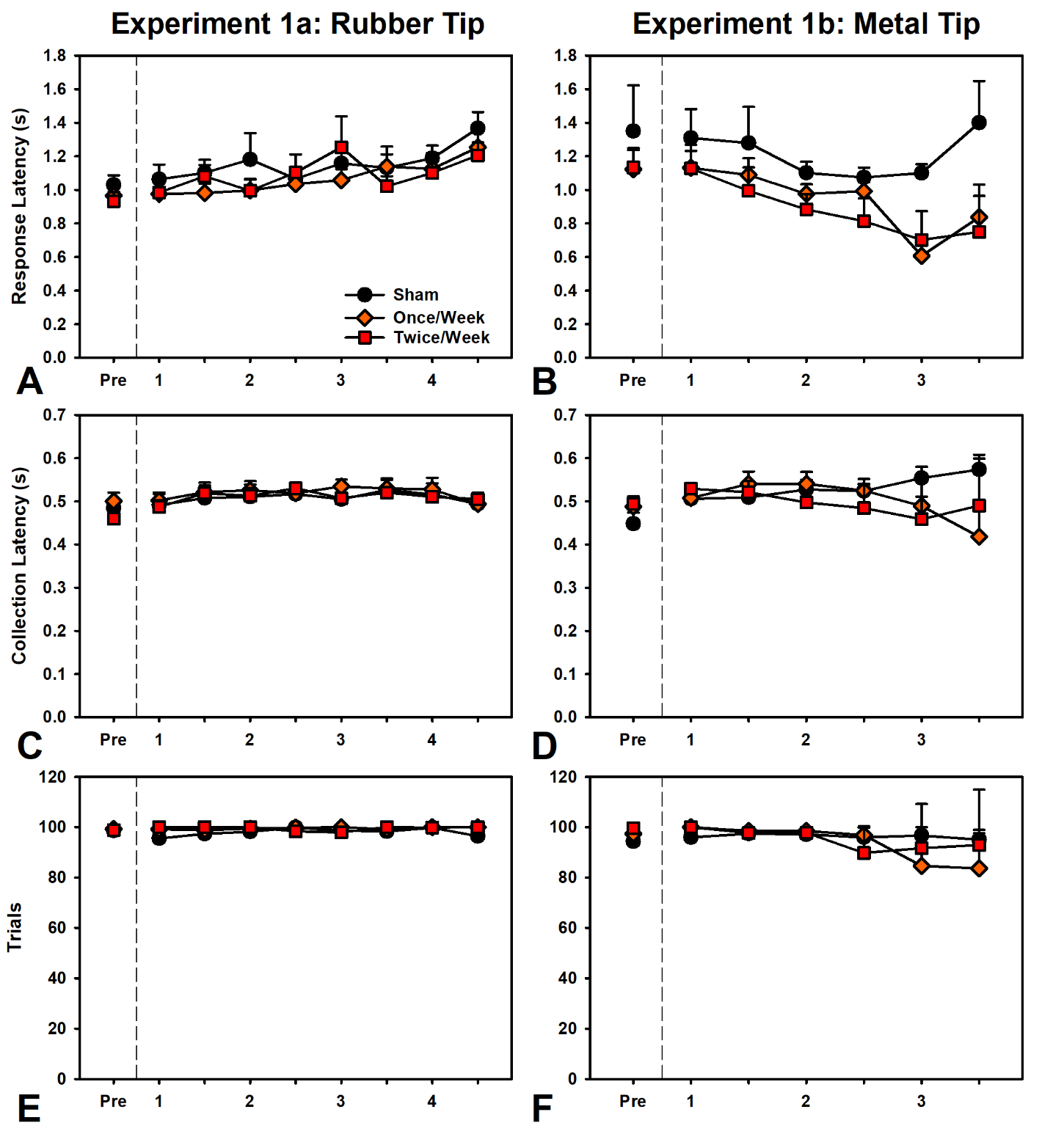


**Figure S1.** Performance on 5CSRT additional variables during Experiment 1a (left panels) and Experiment 1b (right panels). A) There was a small, but significant increase over time in the Twice/Week injury group relative to Sham (*p* = 0.001). B) There was significantly reduced response latency over time for the Once/Week and Twice/Week group compared to Sham (*p* < 0.001; *p* = 0.002). C) There were no group differences in collection latency for the reinforcer. D) Reinforcer collection latencies were also decreased across time in Once/Week and Twice/Week injury groups relative to Sham (*p* < 0.001; *p* < 0.001). E) There were no group differences in total trials. F) Total trials completed was lower across time in both injury groups compared to Sham (*p* < 0.001; *p* < 0.001).


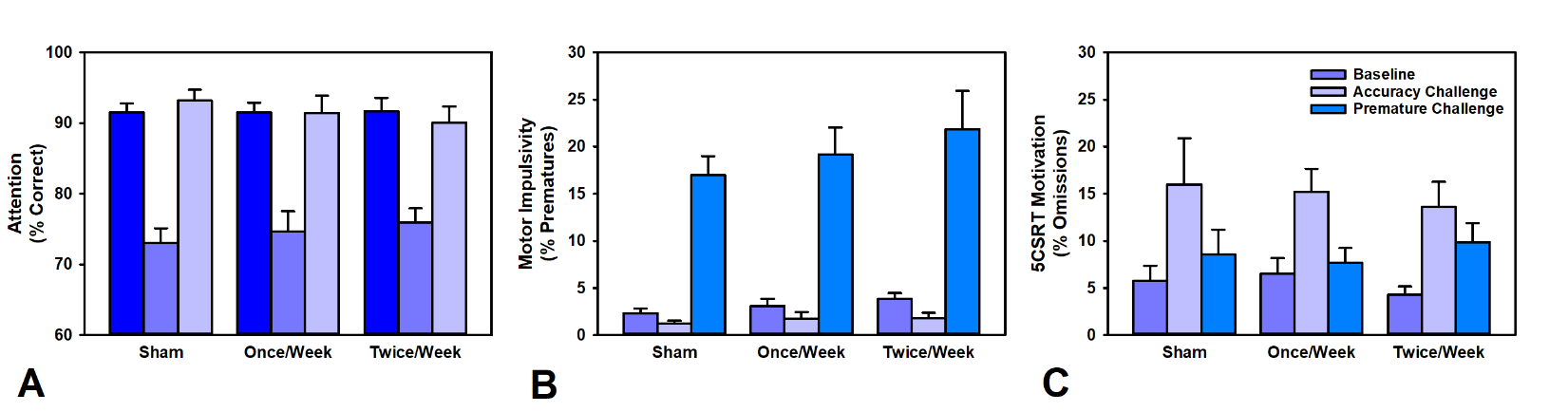


**Figure S2.** Accuracy (short stimulus duration) and impulsivity (long ITI) challenges after Experiment 1a. A) Accuracy was reduced during the accuracy challenge, but there were no group differences. B) Prematures were increased during the premature challenge, but there were no group differences. C) Omissions were elevated during the accuracy challenge, but there were no group differences.


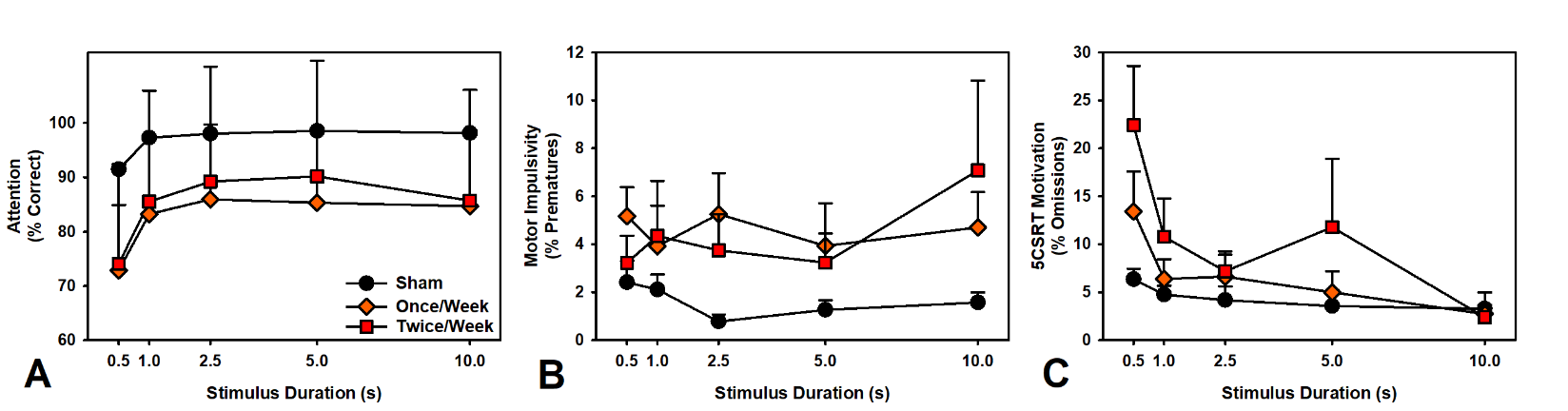


**Figure S3.** Effects of manipulating stimulus duration on performance at the conclusion of Experiment 1b. A) Group differences persisted through changes in stimulus duration, with both injured groups displaying impairment relative to Sham (Once/Week: *p* = 0.002, Twice/Week: *p* = 0.026). B) Overall group differences lost statistical significance after increasing the stimulus duration (*p* = 0.075). C) Overall group differences lost statistical significance after increasing the stimulus duration (*p* = 0.249). Data are means +/- SEM.

| **Experiment 1a - Cushioned Tip** | | **DF** | ***F*** | ***p*** |  | **β** | ***t*** | ***p*** |
| --- | --- | --- | --- | --- | --- | --- | --- | --- |
| Accuracy | Group | 2,30.7 | 0.64 | 0.532 |  |  |  |  |
|  | Week | 1,1011.1 | 51.28 | **<0.001** |  |  |  |  |
|  | Group X Week | 2,1011.1 | 1.44 | 0.237 |  |  |  |  |
| Prematures | Group | 2,33.5 | 0.57 | 0.571 |  |  |  |  |
|  | Week | 1,1011.1 | 24.98 | **<0.001** |  |  |  |  |
|  | Group X Week | 2,1011.1 | 0.07 | 0.936 |  |  |  |  |
| Omissions | Group | 2,28.5 | 2.08 | 0.144 | Sham v. Once/Wk | -0.03 | -0.70 | 0.483 |
|  | Week | 1,1011.1 | 120.55 | **<0.001** | Sham v. Twice/Wk | -0.11 | -3.01 | **0.003** |
|  | Group X Week | 2,1011.1 | 4.96 | **0.007** | Once/Wk v. Twice/Wk | -0.08 | -2.31 | **0.021** |
| Index | Group | 2,29.3 | 2.82 | 0.076 | Sham v. Once/Wk | 0.00 | -0.04 | 0.970 |
|  | Week | 1,1011.1 | 153.36 | **<0.001** | Sham v. Twice/Wk | 0.08 | 2.36 | **0.019** |
|  | Group X Week | 2,1011.1 | 3.77 | **0.023** | Once/Wk v. Twice/Wk | 0.08 | 2.40 | **0.017** |
| Trials | Group | 2,29.7 | 2.56 | 0.094 |  |  |  |  |
|  | Week | 1,1011.1 | 0.95 | 0.331 |  |  |  |  |
|  | Group X Week | 2,1011.1 | 1.39 | 0.250 |  |  |  |  |
| Response Latency | Group | 2,30.6 | 4.43 | **0.020** | Sham v. Once/Wk | -0.01 | -0.22 | 0.828 |
|  | Week | 1,1011.1 | 80.11 | **<0.001** | Sham v. Twice/Wk | 0.14 | 3.45 | **0.001** |
|  | Group X Week | 2,1011.1 | 8.46 | **<0.001** | Once/Wk v. Twice/Wk | 0.15 | 3.67 | **<0.001** |
| Collection Latency | Group | 2.30.1 | 0.59 | 0.561 |  |  |  |  |
|  | Week | 1,1011.1 | 163.76 | **<0.001** |  |  |  |  |
|  | Group X Week | 2,1011.1 | 1.41 | 0.244 |  |  |  |  |

**Table S1.** Statistics for Experiment 1a on the 5CSRT.

| **Experiment 1b - Metal Tip** | | **DF** | ***F*** | ***p*** |  | **β** | ***t*** | ***p*** |
| --- | --- | --- | --- | --- | --- | --- | --- | --- |
| Accuracy | Group | 2,26.6 | 15.88 | **<0.001** | Sham v. Once/Wk | -0.54 | -10.05 | **<0.001** |
|  | Week | 1,777.5 | 277.19 | **<0.001** | Sham v. Twice/Wk | -0.48 | -8.39 | **<0.001** |
|  | Group X Week | 2,776.7 | 60.00 | **<0.001** | Once/Wk v. Twice/Wk | 0.06 | 1.00 | 0.318 |
| Prematures | Group | 2,28.4 | 12.65 | **<0.001** | Sham v. Once/Wk | 0.35 | 5.40 | **<0.001** |
|  | Week | 1,783.8 | 54.62 | **<0.001** | Sham v. Twice/Wk | 0.31 | 4.52 | **<0.001** |
|  | Group X Week | 2,781.8 | 17.35 | **<0.001** | Once/Wk v. Twice/Wk | -0.04 | -0.54 | 0.592 |
| Omissions | Group | 2,27.2 | 9.49 | **0.001** | Sham v. Once/Wk | 0.31 | 5.51 | **<0.001** |
|  | Week | 1,778.6 | 51.54 | **<0.001** | Sham v. Twice/Wk | 0.35 | 5.91 | **<0.001** |
|  | Group X Week | 2,777.3 | 22.61 | **<0.001** | Once/Wk v. Twice/Wk | 0.05 | 0.75 | 0.456 |
| Index | Group | 2,27 | 18.92 | **<0.001** | Sham v. Once/Wk | -0.53 | -9.58 | **<0.001** |
|  | Week | 1,778.8 | 209.13 | **<0.001** | Sham v. Twice/Wk | -0.49 | -8.39 | **<0.001** |
|  | Group X Week | 2,777.7 | 56.42 | **<0.001** | Once/Wk v. Twice/Wk | 0.03 | 0.57 | 0.569 |
| Trials | Group | 2,28.1 | 4.21 | **0.025** | Sham v. Once/Wk | -0.26 | -3.99 | **<0.001** |
|  | Week | 1,785.1 | 29.50 | **<0.001** | Sham v. Twice/Wk | -0.21 | -3.09 | **0.002** |
|  | Group X Week | 2,782.8 | 9.01 | **<0.001** | Once/Wk v. Twice/Wk | 0.05 | 0.65 | 0.519 |
| Response Latency | Group | 2,29.1 | 21.14 | **<0.001** | Sham v. Once/Wk | -0.49 | -7.41 | **<0.001** |
|  | Week | 1,795.7 | 9.54 | **0.002** | Sham v. Twice/Wk | -0.42 | -6.02 | **<0.001** |
|  | Group X Week | 2,794.2 | 31.72 | **<0.001** | Once/Wk v. Twice/Wk | 0.07 | 1.04 | 0.299 |
| Collection Latency | Group | 2,26.9 | 7.82 | **0.002** | Sham v. Once/Wk | -0.43 | -8.34 | **<0.001** |
|  | Week | 1,774.9 | 173.70 | **<0.001** | Sham v. Twice/Wk | -0.42 | -7.63 | **<0.001** |
|  | Group X Week | 2,774.3 | 44.35 | **<0.001** | Once/Wk v. Twice/Wk | 0.01 | 0.18 | 0.856 |

**Table S2.** Statistics for Experiment 1b on the 5CSRT.

| **Challenges** |  | **DF** | ***F*** | ***p*** |
| --- | --- | --- | --- | --- |
| Accuracy | Accuracy | 2,26 | 0.60 | 0.554 |
| 0.25s Stim | Prematures | 2,26 | 0.26 | 0.775 |
|  | Omissions | 2,26 | 0.34 | 0.715 |
| Premature | Accuracy | 2,25 | 0.80 | 0.461 |
| 7.5s ITI | Prematures | 2,25 | 0.38 | 0.687 |
|  | Omissions | 2,25 | 0.75 | 0.484 |

**Table S3.** Statistics for Behavioral Challenges.

| **Stimulus Duration Manipulation** | | **DF** | ***F*** | ***p*** |  | **β** | ***t*** | ***p*** |
| --- | --- | --- | --- | --- | --- | --- | --- | --- |
| Accuracy | Group | 2,21 | 7.07 | **0.005** | Once/Wk v. Sham | -1.45 | -3.61 | **0.002** |
|  | Stim. Duration | 1,92 | 1.18 | **0.041** | Twice/Wk v. Sham | -1.15 | -2.40 | **0.026** |
|  | Group x Duration | 1,92 | 0.10 | 0.902 | Once/Wk v. Twice/Wk | 0.30 | 0.62 | 0.541 |
| Prematures | Group | 2,20 | 2.96 | 0.075 |  |  |  |  |
|  | Stim. Duration | 1,89 | 0.69 | 0.979 |  |  |  |  |
|  | Group x Duration | 2,89 | 0.69 | 0.502 |  |  |  |  |
| Omissions | Group | 2,20 | 1.49 | 0.249 |  |  |  |  |
|  | Stim. Duration | 1,89 | 0.73 | 0.374 |  |  |  |  |
|  | Group x Duration | 2,89 | 0.04 | 0.963 |  |  |  |  |

**Table S4.** Statistics for Stimulus Duration Manipulation.

| **Experiment 2 - 5CSRT** | | **Injury Period** | | | **Recovery Period** | | |
| --- | --- | --- | --- | --- | --- | --- | --- |
|  |  | **β** | ***t*** | ***p*** | **β** | ***t*** | ***p*** |
| Accuracy | Injury | -0.99 | -3.98 | **0.001** | -1.57 | -3.47 | **0.004** |
|  | Week | -0.05 | -1.41 | 0.160 | 0.05 | 2.17 | **0.031** |
|  | Injury x Week | -0.51 | -9.15 | **<0.001** | -0.01 | -0.23 | 0.820 |
| Prematures | Injury | 0.02 | 0.11 | 0.915 | 0.02 | 0.06 | 0.955 |
|  | Week | -0.11 | -2.04 | **0.042** | -0.04 | -0.92 | 0.356 |
|  | Injury x Week | 0.00 | 0.07 | 0.948 | -0.01 | -0.18 | 0.860 |
| Omissions | Injury | 0.80 | 3.59 | **0.002** | 1.07 | 3.13 | **0.006** |
|  | Week | 0.06 | 1.71 | 0.087 | 0.02 | 0.59 | 0.554 |
|  | Injury x Week | 0.38 | 7.18 | **<0.001** | 0.02 | 0.46 | 0.643 |
| Index | Injury | -0.96 | -4.18 | **<0.001** | -1.46 | -4.38 | **<0.001** |
|  | Week | -0.01 | -0.20 | 0.841 | 0.03 | 1.45 | 0.148 |
|  | Injury x Week | -0.60 | -13.52 | **<0.001** | -0.05 | -1.74 | 0.082 |
| Trials | Injury | -0.92 | -3.53 | **0.002** | -1.45 | -4.07 | **0.001** |
|  | Week | -0.08 | -2.33 | **0.020** | -0.01 | -0.59 | 0.556 |
|  | Injury x Week | -0.55 | -11.52 | **<0.001** | -0.03 | -1.54 | 0.124 |
| Response Latency | Injury | 1.04 | 3.84 | **0.001** | 0.93 | 3.55 | **0.006** |
|  | Week | 0.01 | 0.20 | 0.839 | -0.05 | -0.88 | 0.382 |
|  | Injury x Week | 0.52 | 8.49 | **<0.001** | 0.00 | -0.04 | 0.966 |
| Collection Latency | Injury | 0.09 | 0.61 | 0.550 | 0.39 | 0.85 | 0.409 |
|  | Week | 0.02 | 0.50 | 0.615 | -0.05 | -0.98 | 0.326 |
|  | Injury x Week | 0.05 | 0.73 | 0.466 | 0.02 | 0.29 | 0.773 |

**Table S5.** Statistics for 5CSRT performance in Experiment 2.

| **Experiment 2 - DDT** | |  | **DF** | ***F*** | ***p*** |  | **β** | ***t*** | ***p*** |
| --- | --- | --- | --- | --- | --- | --- | --- | --- | --- |
| **Injury Period** | k value |  |  |  |  | Injury | -0.30 | -1.57 | 0.133 |
|  |  |  |  |  |  | Week | 0.13 | 2.17 | **0.033** |
|  |  |  |  |  |  | Injury x Week | -0.40 | -4.58 | **<0.001** |
|  | Omissions | Injury | 1,20.62 | 17.18 | **<0.001** |  |  |  |  |
|  |  | Week | 1,1989.33 | 324.93 | **<0.001** |  |  |  |  |
|  |  | Delay | 1,1996.07 | 112.71 | **<0.001** |  |  |  |  |
|  |  | Injury*Week | 1,1988.29 | 137.41 | **<0.001** |  |  |  |  |
|  |  | Injury*Delay | 1,1980.23 | 32.20 | **<0.001** |  |  |  |  |
|  |  | Week*Delay | 1,1979.17 | 70.77 | **<0.001** |  |  |  |  |
|  |  | Injury*Week*Delay | 1,1979.41 | 8.22 | **0.004** | Sham: Week*Delay | 0.09 | 3.82 | **<0.001** |
|  |  |  |  |  |  | CHIMERA: Week*Delay | 0.18 | 8.18 | **<0.001** |
|  | Choice  Latency | Injury | 1,21.14 | 2.82 | 0.108 |  |  |  |  |
|  |  | Week | 1,1932.89 | 111.90 | **<0.001** |  |  |  |  |
|  |  | Delay | 1,1724.34 | 30.35 | **<0.001** |  |  |  |  |
|  |  | Injury*Week | 1,1932.82 | 34.83 | **<0.001** |  |  |  |  |
|  |  | Injury*Delay | 1,1936.91 | 13.63 | **<0.001** |  |  |  |  |
|  |  | Week*Delay | 1,1923.81 | 5.75 | **0.017** |  |  |  |  |
|  |  | Injury*Week*Delay | 1,1924.56 | 7.52 | **0.006** | Sham: Week*Delay | 0.00 | -0.24 | 0.807 |
|  |  |  |  |  |  | CHIMERA: Week*Delay | 0.06 | 3.62 | **<0.001** |
| **Recovery Period** | k value |  |  |  |  | Injury | -0.73 | -2.16 | **0.045** |
|  |  |  |  |  |  | Week | -0.03 | -1.06 | 0.296 |
|  |  |  |  |  |  | Injury x Week | 0.08 | 1.77 | 0.081 |
|  | Omissions | Injury | 1,17.87 | 5.77 | **0.027** |  |  |  |  |
|  |  | Week | 1,1407.95 | 3.18 | 0.075 |  |  |  |  |
|  |  | Delay | 1,1421.4 | 190.92 | **<0.001** |  |  |  |  |
|  |  | Injury*Week | 1,1407.95 | 2.53 | 0.112 |  |  |  |  |
|  |  | Injury*Delay | 1,1411.57 | 79.62 | **<0.001** | Sham: Delay | 0.14 | 4.99 | **<0.001** |
|  |  |  |  |  |  | CHIMERA: Delay | 0.46 | 15.62 | **<0.001** |
|  |  | Week*Delay | 1,1407.92 | 12.57 | **<0.001** |  |  |  |  |
|  |  | Injury*Week*Delay | 1,1407.92 | 1.95 | 0.163 |  |  |  |  |
|  | Choice  Latency | Injury | 1,17.63 | 1.48 | 0.240 |  |  |  |  |
|  |  | Week | 1,1367.89 | 1.22 | 0.270 |  |  |  |  |
|  |  | Delay | 1,1376.21 | 87.86 | **<0.001** |  |  |  |  |
|  |  | Injury*Week | 1,1367.9 | 4.69 | **0.031** | Sham: Week | 0.05 | 2.37 | **0.018** |
|  |  |  |  |  |  | CHIMERA: Week | -0.02 | -0.73 | 0.464 |
|  |  | Injury*Delay | 1,1376.03 | 92.56 | **<0.001** | Sham: Delay | 0.01 | 0.50 | 0.619 |
|  |  |  |  |  |  | CHIMERA: Delay | 0.31 | 13.41 | **<0.001** |
|  |  | Week*Delay | 1,1367.93 | 4.35 | **0.037** |  |  |  |  |
|  |  | Injury*Week*Delay | 1,1367.97 | 0.72 | 0.397 |  |  |  |  |

**Table S6.** Statistics for DDT performance in Experiment 2.

| **Experiment 1** |  | **DF** | ***F*** | ***p*** |  |  | **β** | ***t*** | ***p*** |
| --- | --- | --- | --- | --- | --- | --- | --- | --- | --- |
| **IBA1  Counts** | Group | 2,24.46 | 0.81 | 0.459 | PFC | Once/Wk v. Sham | -0.31 | -0.76 | 0.457 |
|  | ROI | 4,340.61 | 28.44 | **<0.001** |  | Twice/Wk v. Sham | 0.09 | 0.18 | 0.855 |
|  | Group*ROI | 8,340.23 | 3.70 | **<0.001** |  | Once/Wk v. Twice/Wk | 0.40 | 0.84 | 0.410 |
|  |  |  |  |  | OFC | Once/Wk v. Sham | 0.80 | 2.04 | 0.053 |
|  |  |  |  |  |  | Twice/Wk v. Sham | -0.02 | -0.06 | 0.954 |
|  |  |  |  |  |  | Once/Wk v. Twice/Wk | -0.83 | -1.97 | 0.062 |
|  |  |  |  |  | dSTR | Once/Wk v. Sham | -0.21 | -0.53 | 0.600 |
|  |  |  |  |  |  | Twice/Wk v. Sham | -0.94 | -2.04 | 0.053 |
|  |  |  |  |  |  | Once/Wk v. Twice/Wk | -0.73 | -1.58 | 0.128 |
|  |  |  |  |  | NAc-Core | Once/Wk v. Sham | 0.31 | 0.75 | 0.459 |
|  |  |  |  |  |  | Twice/Wk v. Sham | 0.14 | 0.30 | 0.765 |
|  |  |  |  |  |  | Once/Wk v. Twice/Wk | -0.17 | -0.36 | 0.721 |
|  |  |  |  |  | NAc-Shell | Once/Wk v. Sham | 0.47 | 1.11 | 0.282 |
|  |  |  |  |  |  | Twice/Wk v. Sham | 0.22 | 0.43 | 0.669 |
|  |  |  |  |  |  | Once/Wk v. Twice/Wk | -0.25 | -0.51 | 0.618 |
| **IBA1  Phenotype** | Group | 2, 28.08 | 1.85 | 0.175 | PFC | Once/Wk v. Sham | -1.44 | -2.74 | **0.006** |
|  | ROI | 4, 2408.56 | 7.03 | **<0.001** | Interaction | Twice/Wk v. Sham | 1.22 | 1.96 | 0.051 |
|  | Circularity | 1, 2406.87 | 2212.25 | **<0.001** |  | Once/Wk v. Twice/Wk | 2.66 | 4.18 | **<0.001** |
|  | Group*ROI | 8, 2408.55 | 3.21 | **0.001** | OFC | Once/Wk v. Sham | -0.95 | -1.70 | 0.089 |
|  | Group*Circ. | 2, 2406.87 | 9.21 | **<0.001** | Interaction | Twice/Wk v. Sham | 1.51 | 2.29 | **0.023** |
|  | ROI*Circ. | 4, 2406.87 | 11.97 | **<0.001** |  | Once/Wk v. Twice/Wk | 2.46 | 3.65 | **<0.001** |
|  | Group*ROI*Circ | 8, 2406.87 | 2.86 | **0.004** | dSTR | Once/Wk v. Sham | 0.46 | 0.98 | 0.328 |
|  |  |  |  |  | Interaction | Twice/Wk v. Sham | 0.09 | 0.17 | 0.862 |
|  |  |  |  |  |  | Once/Wk v. Twice/Wk | -0.37 | -0.70 | 0.487 |
|  |  |  |  |  | NAc | Once/Wk v. Sham | 2.04 | 3.61 | **<0.001** |
|  |  |  |  |  | Interaction | Twice/Wk v. Sham | 2.93 | 4.52 | **<0.001** |
|  |  |  |  |  |  | Once/Wk v. Twice/Wk | 0.89 | 1.40 | 0.164 |
| **GFAP  Counts** | Group | 2,27.06 | 4.45 | **0.021** | Overall | Once/Wk v. Sham | 0.07 | 0.34 | 0.737 |
|  | ROI | 4,352.18 | 160.64 | **<0.001** |  | Twice/Wk v. Sham | 0.42 | 2.09 | **0.039** |
|  | Group*ROI | 8,351.76 | 1.41 | 0.190 |  | Once/Wk v. Twice/Wk | 0.35 | 1.73 | 0.087 |
| **GFAP  % Area** | Group | 2,26.41 | 2.50 | 0.1422 | PFC | Once/Wk v. Sham | -0.44 | -1.10 | 0.280 |
|  | ROI | 4,354.48 | 25.99 | **<0.001** |  | Twice/Wk v. Sham | 0.36 | 0.87 | 0.392 |
|  | Group*ROI | 8,354.14 | 9.86 | **<0.001** |  | Once/Wk v. Twice/Wk | 0.80 | 1.94 | 0.063 |
|  |  |  |  |  | OFC | Once/Wk v. Sham | -0.32 | -0.73 | 0.475 |
|  |  |  |  |  |  | Twice/Wk v. Sham | 0.48 | 1.08 | 0.291 |
|  |  |  |  |  |  | Once/Wk v. Twice/Wk | 0.80 | 1.83 | 0.079 |
|  |  |  |  |  | NAc | Once/Wk v. Sham | -1.15 | -3.35 | **<0.001** |
|  |  |  |  |  |  | Twice/Wk v. Sham | 0.43 | 1.27 | 0.218 |
|  |  |  |  |  |  | Once/Wk v. Twice/Wk | 1.58 | 4.38 | **<0.001** |
|  |  |  |  |  | dSTR | Once/Wk v. Sham | -0.31 | -0.69 | 0.496 |
|  |  |  |  |  |  | Twice/Wk v. Sham | -1.15 | -2.85 | **0.010** |
|  |  |  |  |  |  | Once/Wk v. Twice/Wk | -0.84 | -1.86 | 0.077 |

**Table S7.** Statistics for histology, Experiment 1.

| **Experiment 2** |  | **DF** | ***F*** | ***p*** |
| --- | --- | --- | --- | --- |
| **IBA1 Counts** | Group | 1,18.03 | 0.22 | 0.642 |
|  | ROI | 4,370.1 | 10.64 | **<0.001** |
|  | Group*ROI | 4,370.1 | 2.36 | 0.053 |
| **IBA1 Phenotype** | Group | 1,20.63 | 0.37 | 0.549 |
|  | ROI | 4,2350.05 | 28.19 | **<0.001** |
|  | Circularity | 1,2350.01 | 3758.06 | **<0.001** |
|  | Group*ROI | 4,2350.05 | 4.74 | **0.001** |
|  | Group*Circularity | 1,2350.01 | 0.28 | 0.595 |
|  | ROI*Circularity | 4,2350.01 | 0.31 | 0.870 |
|  | Group*ROI*Circ. | 4,2350.01 | 1.21 | 0.305 |
| **GFAP Counts** | Group | 1,17.94 | 1.38 | 0.255 |
|  | ROI | 4,364.28 | 97.75 | **<0.001** |
|  | Group*ROI | 4,364.28 | 0.41 | 0.801 |
| **GFAP % Area** | Group | 1,17.95 | 0.61 | 0.446 |
|  | ROI | 4,364.14 | 23.40 | **<0.001** |
|  | Group*ROI | 4,364.14 | 0.10 | 0.981 |

**Table S8.** Statistics for histology, Experiment 2.

|  |  |  |  |  |  |  |
| --- | --- | --- | --- | --- | --- | --- |
|  | **Experiment 1** | | | **Experiment 2** | | |
|  | **β** | ***t*** | ***p*** | **β** | ***t*** | ***p*** |
| 5CSRT Index | -0.35 | -6.03 | **<0.001** | -0.32 | -2.97 | **0.005** |
| 5CSRT Accuracy | -0.36 | -6.26 | **<0.001** | -0.16 | -1.23 | 0.225 |
| 5CSRT Prematures | 0.20 | 3.34 | **0.001** | -0.33 | -2.99 | **0.004** |
| 5CSRT Omissions | 0.27 | 4.78 | **<0.001** | 0.28 | 3.05 | **0.004** |
| DDT k |  |  |  | -0.07 | -0.73 | 0.468 |
| DDT Omissions |  |  |  | 0.29 | 3.01 | **0.004** |

**Table S9.** Statistics for LRR predicting behavior, Experiment 2.
